# Coagulation Parameters in Human Immunodeficiency Virus Infected Patients: A Systematic Review and Meta-Analysis

**DOI:** 10.1155/2022/6782595

**Published:** 2022-04-21

**Authors:** Solomon Getawa, Tiruneh Adane

**Affiliations:** Department of Hematology and Immunohematology, School of Biomedical and Laboratory Sciences, College of Medicine and Health Sciences, University of Gondar, Gondar, Ethiopia

## Abstract

**Background:**

Coagulation abnormalities are common complications of human immunodeficiency virus (HIV) infection. Highly active antiretroviral treatment (HAART) decreased the mortality of HIV but increased coagulopathies. HIV-related thrombocytopenia, prolonged prothrombin time (PT), activated partial thromboplastin time (APTT), and high D-dimer level commonly manifested in patients with HIV. Thus, this study is aimed to compare coagulation parameters of HAART-treated and HAART-naïve HIV-infected patients with HIV-seronegative controls.

**Methods:**

A systematic literature search was conducted using the databases PubMed/MEDLINE, Embase, Web of Science, and Google Scholar of studies published until July 2021. The primary outcome of interest was determining the pooled mean difference of coagulation parameters between HIV-infected patients and seronegative controls. The Joana Briggs Institute (JBI) critical appraisal tool was used for quality appraisal. Statistical analyses were performed using Stata11.0 software. The statistical results were expressed as the effect measured by standardized mean difference (SMD) with their related 95% confidence interval (CI).

**Results:**

A total of 7,498 participants (1,144 HAART-naïve patients and 2,270 HAART-treated HIV-infected patients and 3,584 HIV-seronegative controls) from 18 studies were included. HIV-infected patients (both on HAART and HAART-naive) exhibited significantly higher levels of PT than HIV-seronegative controls (SMD = 0.66; 95% CI: 0.53–0.80 and SMD = 1.13; 95% CI: 0.60–2.0, respectively). The value of APTT was significantly higher in patients with HIV on HAART than in seronegative controls. However, the values of PLT count, APTT, and fibrinogen level were significantly higher in seronegative controls. Besides, the level of fibrinogen was significantly higher in HAART-treated than treatment-naïve patients (SMD = 0.32; 95%CI: 0.08, 0.57). Moreover, the level of APTT and PT had no statistical difference between HAART and HAART-naïve HIV-infected patients.

**Conclusions:**

This study identified that HIV-infected patients are more likely to develop coagulation abnormalities than HIV-seronegative controls. Therefore, coagulation parameters should be assessed regularly to prevent and monitor coagulation disorders in HIV-infected patients.

## 1. Introduction

HIV infection is an illness with protean manifestations including opportunistic infections, autoimmune disorders, and solid and hematologic malignancies. Cytopenia due to marrow dysplasia, infections, immune destruction, or drug effects, and hemostatic disorders are the most common manifestations of HIV infection [1]. Hemostatic abnormalities occur frequently in patients with HIV. In HIV infection, hemostatic disorders occur as a result of acquired deficiency of anticoagulant proteins such as protein C and protein S [2–4], heparin cofactor II [5],and increased concentrations of coagulation and fibrinolytic markers [6–8]. HIV infection and HAART impair liver function by inducing hepatotoxicity that diminishes the function and synthesis of coagulation factors [9].

Chronic immune activation and inflammatory state in both untreated and treated HIV infections can result in abnormal hemostatic changes. Immune-mediated destruction of platelets by antibodies, impaired megakaryocytes, hypersplenism, opportunistic infections, malignancy, and toxic and myelosuppressive effects of HIV medications may lead to thrombocytopenia and alter the hemostatic system in patients with HIV [10, 11]. Alterations in vascular function and endothelial cell dysfunction due to chronic inflammation have been reported in HIV infection [12, 13]. Damaged endothelial cells can initiate a coagulation cascade and increase the levels of von Willebrand factor (vWf), which may enhance platelet adhesion and clot formation [2, 8].

In HIV infection, markers related to inflammation such as IL-6 and C-reactive protein and coagulation including tissue factor expression, Factor VIII, thrombin, fibrinogen, and D-dimer levels are increased and cause thrombotic risk and mortality [14]. The morbidity and mortality of patients with HIV infection due to nonacquired immunodeficiency syndrome (AIDS) related causes such as cardiovascular disease has increased recently [15]. Inflammation, platelet activation, and alterations in the coagulation pathway may contribute to microvascular dysfunction [16–18]. Plasma levels of the coagulation biomarker D-dimer are elevated among treated HIV-positive patients compared to uninfected controls [4, 19, 20]. Prolonged APTT, due to the production of a lupus anticoagulant and anticardiolipin antibodies, also occurred in patients with HIV [21].

Evidence suggests that HIV replication may upregulate the tissue factor pathway via innate immune activation. Untreated HIV replication leads to short-term increases in some procoagulants as well as decreases in key anticoagulants [22, 23]. Therefore, the main aim of this study is to compare coagulation parameters in HAART-treated and untreated HIV-infected patients and HIV-seronegative controls.

## 2. Methods

### 2.1. Information Sources, and Search Strategy

We conducted a systematic and comprehensive search to identify eligible studies using PubMed/MEDLINE, Embase, Web of Science, and Google Scholar databases of studies published until July 2021. The search strategy was based on the combinations of key words and medical subject heading (MeSH) terms using Boolean operators like “OR” or “AND.” The search terms included “hemostatic parameter,” OR “coagulation parameter,” OR “coagulation profile,” OR “prothrombin time,” OR “activated partial thromboplastin time,” OR “platelet count,” OR “D-dimer,” OR “fibrinogen” AND “HIV,” OR “HIV/AIDS” (see Supplementary File Table S1). The reference lists of selected studies were also checked for identifying additional eligible studies. The Preferred Reporting Items for Systematic Reviews and Meta-Analyses (PRISMA-P 2015) guideline [24] was used to report the result of the study (see Supplementary File Table S2).

### 2.2. Eligibility Criteria

The inclusion criteria included the following: (1) study population: all HIV-infected patients who were taking HAART or HAART naive; (2) study design: cross-sectional, prospective/retrospective cohort, and case-control study; (3) if they were the reports of original research and report data on the coagulation parameters including PT, APTT, fibrinogen, D-dimer, and PLT among HIV/AIDS-infected patients; and (4) were published in English language. The exclusion criteria included (1) HIV-infected patients with comorbidities like tuberculosis and malaria; (2) studies without reporting coagulation parameters; and (3) systematic reviews, meta-analyses, editorials, and other forms not presenting original data.

### 2.3. Outcomes of Interest

The primary outcome of interest is to determine the pooled mean difference of PT, APTT, fibrinogen, D-dimer, and PLT count between HIV-infected patients on HAART, HAART-naïve, and HIV-seronegative controls.

### 2.4. Study Selection

The results of the initial search strategy were imported and managed using the EndNoteX7.0 software to remove duplicate articles. Eligible studies are first screened by title and abstract to exclude apparently irrelevant articles by the authors (SG and TA) independently. The remaining full-text articles were subjected to further screening based on inclusion and exclusion criteria by the authors independently, and any disagreement was resolved through discussion.

### 2.5. Data Extraction

Two reviewers independently extracted the following data from the included articles: author's name, study setting, year of publication, sample size, study population (on HAART, HAART-naive, and seronegative controls), and coagulation parameters (PLT, PT, fibrinogen, D-dimer, and APTT). Continuous variables were presented as mean ± standard deviation (SD). If variables were represented by median and interquartile range (IQR), we used the Excel software to convert them to the form of mean ± SD as recommended by Hozo et al. [25].

### 2.6. Methodological Quality Assessment

The Joana Briggs Institute (JBI) critical appraisal checklist for simple prevalence was used to score the methodological quality of included studies. The checklist consists of 9 items, each of which could be answered “yes” (1 point) or “not appropriate and not reported” (0 point). Total scores ranged between 0 and 9, and studies fulfilling 50% and above of quality assessment were included for analysis [26] (see Supplementary File Table S3).

### 2.7. Data Analysis

The data were meta-analyzed using STATA 1\\\1.0 software. Statistical results were expressed in the form of standard mean deference (SMD) with its corresponding 95% CI. Heterogeneity analysis of the included studies was carried out by Higgin's *I*^2^ statistics to determine whether the fixed-effect or random-effect model was applied [27]. The random-effect model was applied due to the presence of significant heterogeneity in the included studies. Publication bias was examined using Egger's regression test statistics and visual inspection of funnel plots for symmetry [28, 29]. A *p* value of less than 0.05 was considered statistically significant.

## 3. Results

### 3.1. Identified Studies

**Figure 1:**
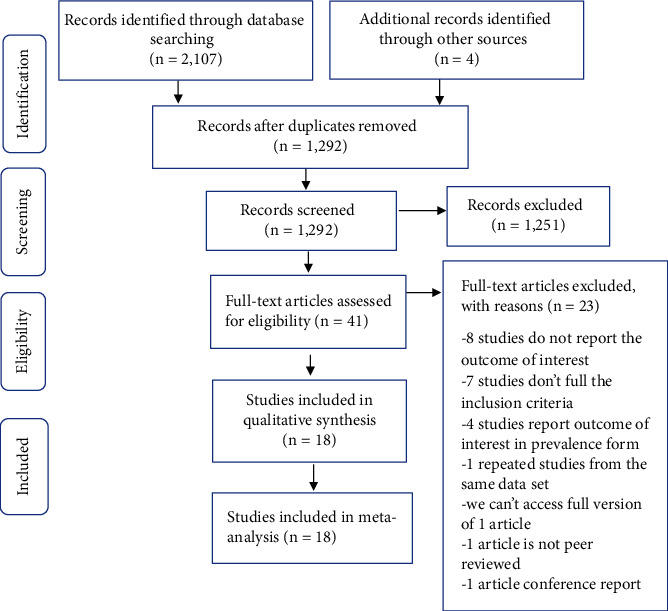
Flow chart describing the selection of studies.

Using different databases and search strategies, 2,111 articles related to coagulation parameters in HIV-infected patients were identified. After removing duplicate articles, 1,292 articles were obtained. Additionally, 1,251 irrelevant articles were excluded by reading their titles and abstracts. The remaining 41 full-text articles were assessed for eligibility and 23 of them were excluded with reason. Finally, 18 articles were selected for qualitative and quantitative analyses (Figure 1).

### 3.2. Description of Selected Studies

A total of 7,498 participants (2,270 on HAART and 1,144 HAART-naïve HIV-infected patients and 3,584 HIV-seronegative controls) were used to analyze and compare coagulation parameters (PT, APTT, fibrinogen, D-dimer, and PLT) in those participants. A total of 18 studies were included; of them 9 studies had been conducted in Nigeria [30–38], 2 from India [39, 40], and the remaining 7 were from Sudan [41], Kenya [42], Ghana [43], Iran [44], Brazil [45], Netherland [46], and Denmark [47]. The included studies had case-control, comparative cross-sectional, and prospective cohort in their study design. The minimum sample size was 90 participants from Nigeria [32] and the maximum was 3891 participants from Denmark [47] (Table 1).

### 3.3. Coagulation Parameters between HIV Patients and Seronegative Controls

**Figure 2:**
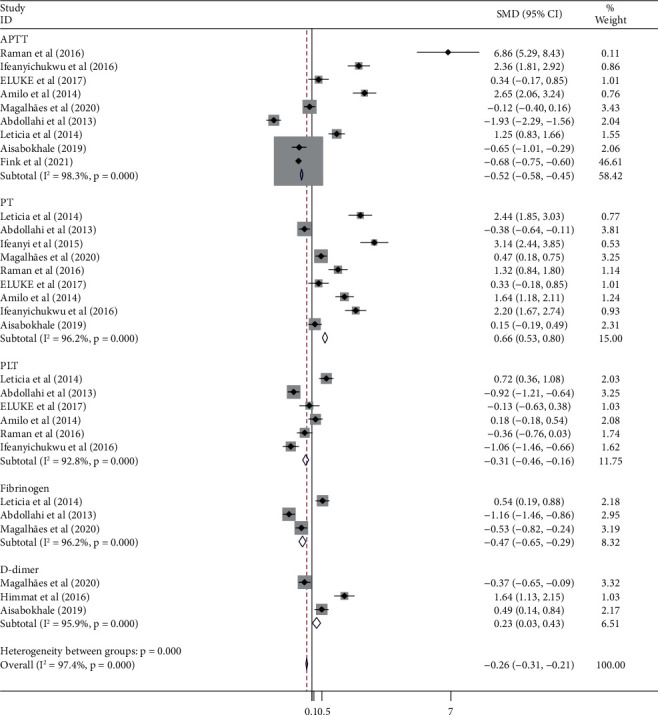
Forest plot displaying a comparison of coagulation parameters between HAART-treated HIV-infected patients and seronegative controls.

We did a meta-analysis on APTT, PLT, fibrinogen, D-dimer, and PT coagulation parameters which were involved in 9, 6, 3, 3, and 9 studies between HIV-infected patients on HAART and seronegative controls. Accordingly, PT and D-dimer values were significantly higher in HIV-infected patients on HAART treatment than seronegative controls (SMD = 0.66; 95% CI: 0.53, 0.80, *I*^2^ = 96.2% and SMD = 0.23; 95% CI: 0.03, 0.423, *I*^2^ = 95.9%, respectively). However, the values of PLT count, APTT, and fibrinogen levels were significantly higher in seronegative controls than in HIV-infected patients on HAART treatment (Figure 2).

**Figure 3:**
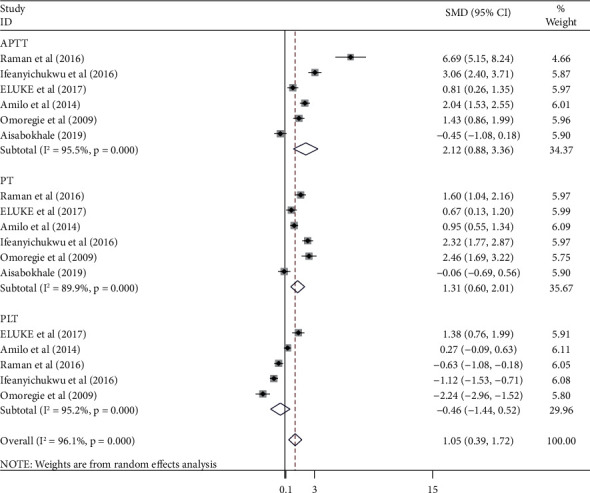
Forest plot showing the comparison of the APTT, PT, and PLT values between HAART-naive HIV-infected patients and seronegative controls.

Also, we compared the APTT, PLT, and PT values which were available in 6, 5, and 6 studies, respectively, but no studies were found to compare fibrinogen and D-dimer levels between HAART-naïve HIV-infected patients and HIV-seronegative controls. Notably, there were significantly higher APTT (SMD = 2.12; 95% CI: 0.88, 3.36, *I*^2^ = 95.5%) and PT (SMD = 1.31; 95% CI: 0.60, 2.01, *I*^2^ = 89.9%) values in HAART-naïve HIV-infected patients than in HIV-seronegative controls. However, there was no significant difference in PLT value (SMD = −0.46; 95% CI: −1.44, 0.52, *I*^2^ = 95.2%) between the two groups (Figure 3).

### 3.4. Coagulation Parameters between HAART and HAART-Naïve Patients

**Figure 4:**
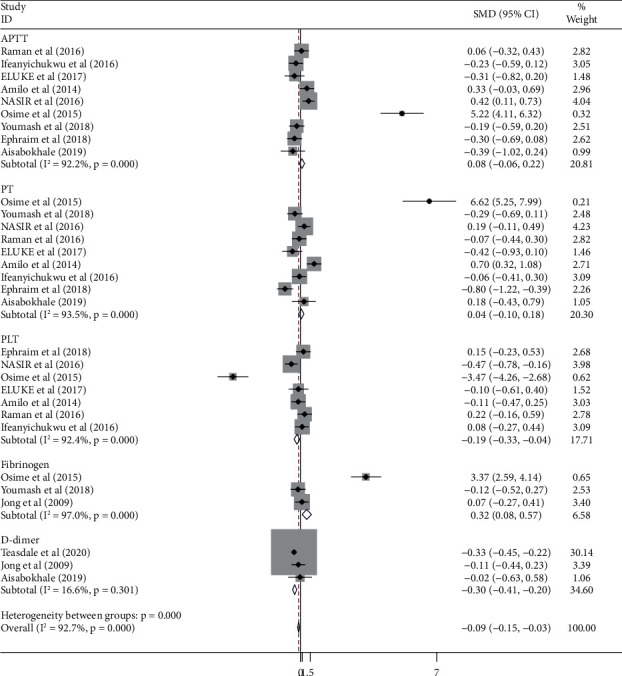
Forest plot showing comparison of coagulation parameters between HAART and HAART- naïve HIV-infected patients.

A total of 9, 7, 3, 3, and 9 studies were used to compare the value of APTT, PLT, fibrinogen, D-dimer and PT, respectively, between HAART and HAART-naïve HIV-infected patients. Accordingly, fibrinogen level was significantly higher in HAART-treated HIV-infected patients than in treatment-naïve patients (SMD = 0.32; 95% CI: 0.08, 0.57). The platelet count and the level of D-dimer were significantly lower in HAART-treated patients than in HAART-naïve patients. However, there is not a significant difference in APTT and PT value between HAART and HAART-naïve patients (Figure 4).

### 3.5. Publication Bias

**Figure 5:**
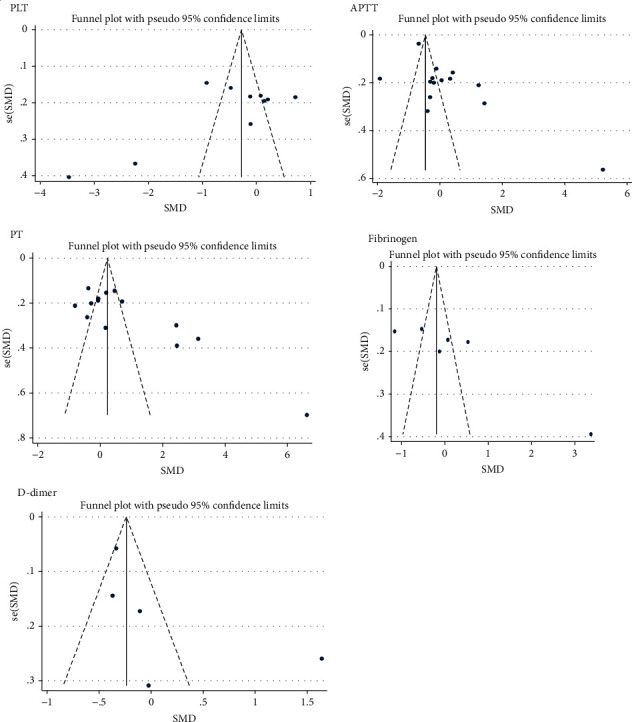
Funnel plot of included studies comparing coagulation parameters among HIV-infected patients.

Included studies comparing coagulation parameters were assessed for publication bias by the Egger's test and visually by the funnel plot. Studies included for comparing PLT count and D-dimer level among HIV-infected patients does not have a significant publication bias. However, studies included for comparing APTT, PT, and fibrinogen values among HIV-infected patients showed a significant publication bias (Table 2 and Figure 5).

## 4. Discussion

HIV infections are characterized by acute and chronic inflammation, leading to cellular and protein changes that can affect the hemostatic system. HIV-associated endothelial dysfunction, thrombocytopenia, presence of anticardiolipin antibody, activation of coagulation factors, and liver disease are the possible causes for the occurrence of coagulation disorders in HIV patients [39, 48, 49]. This may increase the risk of developing arterial thrombosis and venous thromboembolism in both treated and untreated HIV-infected patients [17, 50, 51].

The findings of this review showed that the PT value was significantly higher in HIV-infected patients (both on HAART and HAART-naive) than in HIV-seronegative controls (SMD = 0.66; 95% CI: 0.53, 0.80 and SMD = 1.31; 95% CI: 0.60, 2.01, respectively). The value of APTT was also significantly higher in patients with HIV compared to HIV-seronegative controls. The finding was in agreement with the studies done in Anambra State of Nigeria [34] and Tehran, Iran [44], which reported that the PT and APTT values were higher in HIV-infected patients. Similarly, studies conducted in Jimma and Gondar town of Ethiopia [52, 53], India [40], and the Netherlands [46] also found significantly prolonged PT and APTT values in HIV-infected individuals than in seronegative controls. HIV infection has been associated with endothelial damage, liver disease, and increment of predisposing factors for hypercoagulable state such as presence of anti-cardiolipin antibodies and lupus anticoagulant, and deficiencies of protein C, protein S, heparin cofactor II, and antithrombins, which cause activation and consumption of coagulation factors that can affect the PT and APTT value, which might be the possible reason for the high PT and APTT values among HIV-infected individuals [12, 54–56]. The HAART medications themselves and hepatic damage by the virus may lead to decreased production of coagulation factors causing coagulation abnormalities [39].

This study revealed that there was no significant difference in APTT (SMD = 0.34; 95% CI: −0.16–0.85) and PT (SMD = 0.41; 95% CI: −0.15–0.98) values between HAART-naive HIV-infected individuals as compared to HIV-infected individuals who were taking HAART. This finding was similar to previous single centered studies by Raman et al. who reported that there was no significant difference in APTT and PT value between HAART and HAART-naïve HIV-infected patients [39]. A study by Seyoum et al. in Gondar, Ethiopia, reported that the APTT value of HAART-naive study subjects and HAART groups was not statistically significant (*p*=0.074) [53]. Thus, HAART or liver damage by HAART may not be the actual cause for prolonged PT and APTT in HIV-positive cases. Causes like endothelial dysfunction and hypercoagulable states predisposing to thrombosis lead to the consumption of coagulation factors causing prolonged PT and APTT [39]. However, previous studies reported that the values of APTT and PT were significantly higher in HAART-naïve HIV-infected patients [36, 43, 57]. HAART treatment reduces the viral load level, endothelial dysfunction, and abnormal coagulation activation [58] and also it reduces the production of lupus anticoagulant [59], which may be the possible reason for low APTT and PT values in HAART-treated HIV patients.

We found that the difference in PLT count between HAART-naïve HIV-infected patients and seronegative controls was insignificant. This finding was in concurrence with previous findings by Tagoe and Asantewaa [60] in Ghana which report insignificant difference (*p*=0.111) in the mean PLT count between HIV-positive and HIV-seronegative individuals. However, the PLT count was significantly lower in HIV-infected individuals on HAART compared to seronegative controls. Similarly, studies done by Raman et al. [39] and Omoregie et al. [37] compared low platelet count in HIV-infected individuals to healthy controls. Increased immune-mediated destruction of platelet, presence of antiplatelet specific antibodies, and direct infection of megakaryocytes by HIV, thrombotic thrombocytopenic purpura, impaired hematopoiesis [61, 62] and damaging of the liver by the HIV might be the cause for decreased platelet in HIV-infected patients [63, 64]. This may affect the normal hemostasis and predispose the patients to bleeding.

The values of the PLT count were lower in HAART-treated HIV-infected patients than HAART-naïve patients. However, a previous systematic review showed that the prevalence of thrombocytopenia was higher in treatment-naïve patients than in HIV patients on HAART treatment [65]. HAART treatment reduce disorders of hematopoiesis, opportunistic infections, and immune-mediated destruction of platelet and restoring platelet count in HIV-infected patients [66]. Moreover, HAART-enhanced hematopoietic progenitor cell growth may reduce the viral load by inhibiting viral replication [67]. Thus, the reduction of viral load and immune restoration in HIV-infected patients taking HAART may contribute to a higher platelet count as compared to HAART-naive.

The level of D-dimer was significantly higher in HIV-infected individuals on HAART than in HIV-seronegative controls (SMD = 0.23; 95% CI: 0.03, 0.423). However, it was lower in HAART-treated patients than treatment naïve HIV-infected patients. Several studies confirm that the level of D-dimer is higher in HIV patients than those in hiv-seronegative controls [19, 41, 68]. Hypercoagulable state and activated coagulation system in HIV-infected individuals have been reported in different literature [56, 69]. This elevated D-dimer level indicate an increased reactive fibrinolysis consequent to a hypercoagulable state and thrombotic events [30]. Fibrinogen level was significantly higher in HIV-seronegative controls than in HIV-infected patients. Also, the level of fibrinogen is significantly higher in HAART-treated HIV-infected patients than in HAART-naïve patients (SMD = 0.32; 95% CI: 0.08, 0.57). Similarly, studies reported that HIV patients taking HAART had higher fibrinogen levels than treatment-naïve subjects [70, 71]. These higher levels of fibrinogen levels may contribute to an increased risk of atherosclerosis in HIV-infected subjects [71].

The study has potential limitations. Significant heterogeneity was observed across studies that lead to the conclusion of results being tentative. The inclusion of studies published only in English language may potentially limit the scope of this study.

## 5. Conclusion

The values of APTT, PT, and D-dimer were significantly higher in HIV-infected patients than in HIV-seronegative controls. The platelet count and fibrinogen level are higher in HIV-seronegative controls than in HIV-infected patients. Only fibrinogen level, platelet count, and level of D-dimer show a significant difference in HAART and HAART-naïve HIV-infected patients. Therefore, early screening for coagulation abnormalities irrespective of their treatment status is needed to prevent further complications like cardiovascular diseases in HIV-infected patients.

## Figures and Tables

**Table 1:** Characteristics of the included studies on the coagulation profile of HIV-infected patients.

Author names, publication year	Country	Study design	Sample size			Age groups	Quality score
			HAART	HAART naïve	Seronegative controls		
Leticia et al., 2014 [35]	Nigeria	Case-control	114	—	50	Adult	9
Ephraim et al., 2018 [43]	Ghana	Case-control	65	45	—	All age	8
Raman et al., 2016 [39]	India	Case-control	76	44	40	NR	8
Abdollahi et al., 2013 [44]	Iran	Case-control	114	—	114	Adult	8
Ifeanyichukwu et al., 2016 [34]	Nigeria	Case-control	61	61	60	Adult	9
Osime et al., 2015 [38]	Nigeria	Comparative	50	50	—	NR	7
Ifeanyi and Obeagu, 2015 [33]	Nigeria	Case-control	114	—	50	Adult	6
de Magalhães et al., 2020 [45]	Brazil	Cross-sectional	115	—	88	Elder	9
Omoregie et al., 2009 [37]	Nigeria	Case control	-	70	30	NR	7
Youmash et al., 2018 [40]	India	Cross-sectional	50	50	—	NR	8
Nasir et al., 2016 [36]	Nigeria	Cross-sectional	128	63	—	Adult	9
Chekwube Eluke et al., 2017 [32]	Nigeria	Cross-sectional	30	30	30	NR	7
Aisabokhale et al., 2019 [30]	Nigeria	Comparative	84	12	56	Adult	8
Fink et al., 2021 [47]	Denmark	Case control	936	—	2955	Adult	9
Amilo et al., 2014 [31]	Nigeria	Case-control	60	60	61	Adult	8
Himmat and Gaufri [41]	Sudan	Case-control	50	—	50	NR	8
Jong et al. [46]	Netherland	Comparative	112	48	—	Adult	8
Teasdale et al. [42]	Kenya	Prospective cohort	611	611	—	Adult	9

HAART, highly active antiretroviral therapy; NR, not reported.

**Table 2:** Egger's test.

Parameters	Std_eff	Coef.	Std. err.	t	*P* > *t*	(95% conf. interval)
PLT	Slope	1.12	0.90	1.24	0.25	–0.96, 3.19
	Bias	–7.34	4.57	–1.61	0.14	–17.89, 3.19
APTT	Slope	–0.89	0.20	–4.33	≤0.001	–1.34, –0.44
	Bias	4.87	1.73	2.81	0.01	1.09, 8.63
PT	Slope	–1.62	0.56	–2.87	0.01	–2.84, –0.39
	Bias	9.54	2.72	3.50	≤0.001	3.60, 15.48
Fibrinogen	Slope	–3.14	0.88	–3.55	0.02	–5.58, –0.69
	Bias	16.87	4.90	3.44	0.02	3.27, 30.48
D-Dimer	Slope	–0.59	0.29	–2.01	0.13	–1.52, 0.34
	Bias	3.83	2.63	1.46	0.24	–4.54, 12.20

PLT, platelet; APTT, activated partial thromboplastin time; PT, thromboplastin time.

## Data Availability

The datasets generated and/or analyzed during the current study are available within the article and its supporting materials.

## Abbreviations

AIDS: Acquired immunodeficiency syndrome
HIV: Human immunodeficiency virus
PT: Prothrombin time
plt: Platelet
APTT: Activated partial thromboplastin time
HAART: Highly active antiretroviral therapy.

## Supplementary Materials

Supplementary Materials Table S1: search strategy for PubMed. Table S2: Preferred Reporting Items for Systematic Reviews and Meta-Analyses (PRISMA) checklist. Table S3: quality assessment results of included studies on coagulation parameters of HIV-infected patients.
