# Supplementary material for: Coagulation Parameters in Human Immunodeficiency Virus Infected Patients: A Systematic Review and Meta-Analysis
Source: AIDS Res Treat. 2022 Apr 21;2022:6782595. doi: 10.1155/2022/6782595 (PMC9050251; doi:10.1155/2022/6782595)
Supplement: Supplementary Materials — Table S1: search strategy for PubMed. Table S2: Preferred Reporting Items for Systematic Reviews and Meta-Analyses (PRISMA) checklist. Table S3: quality assessment results of included studies on coagulation parameters of HIV-infected patients. [file 6782595.f1.zip › 6782595.f1/Table S1 Search strategy on pub med HIV.docx]

Table S1 Search strategy for PubMed

| Search’s | **Search terms** | Hits |
| --- | --- | --- |
| #1 | **(((((((hemostatic parameters [Title/Abstract]) OR (coagulation parameters [Title/Abstract])) OR (coagulation profile [Title/Abstract])) OR (prothrombin time [Title/Abstract])) OR (activated partial thromboplastin time [Title/Abstract])) OR (platelet count [Title/Abstract])) OR (D-dimer [Title/Abstract])) OR (fibrinogen [Title/Abstract])** | 98,904 |
| #2 | **(((((((hemostatic parameters [MeSH Terms]) OR (coagulation parameters [MeSH Terms])) OR (coagulation profile [MeSH Terms])) OR (prothrombin time [MeSH Terms])) OR (ctivated partial thromboplastin time [MeSH Terms])) OR (platelet count [MeSH Terms])) OR (D-dimer [MeSH Terms])) OR (fibrinogen [MeSH Terms])** | 99,923 |
| #3 | #1 OR #2 | 151,522 |
| #4 | **((Human immunodeficiency virus [Title/Abstract]) OR (HIV[Title/Abstract])) OR (HIV/AIDS[Title/Abstract])** | 351, 670 |
| #5 | **((Human immunodeficiency virus [MeSH Terms]) OR (HIV [MeSH Terms])) OR (HIV/AIDS [MeSH Terms])** | 134,521 |
| #6 | #4 OR #5 | 363,265 |
| #7 | #3 AND #6 | 1,267 |
