# Supplementary material for: Coagulation Parameters in Human Immunodeficiency Virus Infected Patients: A Systematic Review and Meta-Analysis
Source: AIDS Res Treat. 2022 Apr 21;2022:6782595. doi: 10.1155/2022/6782595 (PMC9050251; doi:10.1155/2022/6782595)
Supplement: Supplementary Materials — Table S1: search strategy for PubMed. Table S2: Preferred Reporting Items for Systematic Reviews and Meta-Analyses (PRISMA) checklist. Table S3: quality assessment results of included studies on coagulation parameters of HIV-infected patients. [file 6782595.f1.zip › 6782595.f1/Table S3 Quality score.docx]

Table S3 Quality assessment result of included studies on coagulation parameters of HIV-infected patients

| Author, year of  Publication | Q1 | Q2 | Q3 | Q4 | Q5 | Q6 | Q7 | Q8 | Q9 | Total score (9%) |
| --- | --- | --- | --- | --- | --- | --- | --- | --- | --- | --- |
| Leticia et al, 2014 ([35](#_ENREF_35)) | Y | Y | Y | Y | Y | Y | Y | Y | Y | 9 |
| Ephraim et al 2018 ([43](#_ENREF_43)) | Y | Y | Y | NA | Y | Y | Y | Y | Y | 8 |
| Raman et al 2016 ([39](#_ENREF_39)) | Y | Y | Y | Y | NR | Y | Y | Y | Y | 8 |
| Abdollahi et al 2013 ([44](#_ENREF_44)) | Y | Y | Y | NR | Y | Y | Y | Y | Y | 8 |
| Ifeanyichukwu et al 2016 ([34](#_ENREF_34)) | Y | Y | Y | Y | Y | Y | Y | Y | Y | 9 |
| Osime et al 2015 ([38](#_ENREF_38)) | Y | Y | Y | NR | NA | Y | Y | Y | Y | 7 |
| Ifeanyi et al 2015 ([33](#_ENREF_33)) | Y | NA | Y | NA | NA | Y | Y | Y | Y | 6 |
| Magalhães et al 2020 ([45](#_ENREF_45)) | Y | Y | Y | Y | Y | Y | Y | Y | Y | 9 |
| Omoregie et al 2009 ([37](#_ENREF_37)) | Y | Y | Y | NR | Y | NA | Y | Y | Y | 7 |
| Youmash et al 2018 ([40](#_ENREF_40)) | Y | Y | Y | NA | Y | Y | Y | Y | Y | 8 |
| Nasir et al 2016 ([36](#_ENREF_36)) | Y | Y | Y | Y | Y | Y | Y | Y | Y | 9 |
| Eluke et al 2017 ([32](#_ENREF_32)) | Y | Y | Y | NA | NR | Y | Y | Y | Y | 7 |
| Aisabokhale et al 2019 ([30](#_ENREF_30)) | Y | Y | Y | NR | Y | Y | Y | Y | Y | 8 |
| Fink et al 2021 ([47](#_ENREF_47)) | Y | Y | Y | Y | Y | Y | Y | Y | Y | 9 |
| Amilo et al 2014 ([31](#_ENREF_31)) | Y | Y | Y | Y | Y | Y | NR | Y | Y | 8 |
| Himmat et al ([41](#_ENREF_41)) | Y | Y | Y | NA | Y | Y | Y | Y | Y | 8 |
| Jong et al ([46](#_ENREF_46)) | Y | Y | Y | NA | Y | Y | Y | Y | Y | 9 |
| Teasdale et al ([42](#_ENREF_42)) | Y | Y | Y | Y | Y | Y | Y | Y | Y | 9 |

**Key:** **Y**= Yes; **NR**= Not reported, **NA**=Not appropriate

**Question codes:**

Q1. Was the sample frame appropriate to address the target population?

Q2. Were study participants sampled in an appropriate way?

Q3. Was the sample size adequate?

Q4. Were the study subjects and the setting described in detail?

Q5. Was the data analysis conducted with sufficient coverage of the identified sample?

Q6. Were valid methods used for the identification of the condition?

Q7. Was the condition measured in a standard, reliable way for all participants?

Q8. Was there appropriate statistical analysis?

Q9. was the response rate adequate, and if not, was the low response rate managed appropriately?
